# Gut microbiota profiles and characterization of cultivable fungal isolates in IBS patients

**DOI:** 10.1007/s00253-021-11264-4

**Published:** 2021-04-10

**Authors:** Piero Sciavilla, Francesco Strati, Monica Di Paola, Monica Modesto, Francesco Vitali, Duccio Cavalieri, Gian Maria Prati, Maura Di Vito, Giovanni Aragona, Carlotta De Filippo, Paola Mattarelli

**Affiliations:** 1 Department of Agriculture and Food Sciences, University of Bologna, Viale Fanin 44, 40127 Bologna, Italy; 2 Laboratory of Mucosal Immunology, Department of Experimental Oncology, European Institute of Oncology, Via Adamello 16, 20139 Milan, Italy; 3 Department of Biology, University of Florence, Via Madonna del Piano 6, 50019, Sesto Fiorentino, Florence, Italy; 4 Institute of Agriculture Biology and Biotechnology, National Research Council (CNR), Via Moruzzi 1, 56124 Pisa, Italy; 5 Department of Gastroenterology and Hepatology, “G da Saliceto” Hospital, Via Taverna 49, 29121 Piacenza, Italy

**Keywords:** Irritable bowel syndrome, Gut microbiota, Gut mycobiota

## Abstract

**Abstract:**

Studies so far conducted on irritable bowel syndrome (IBS) have been focused mainly on the role of gut bacterial dysbiosis in modulating the intestinal permeability, inflammation, and motility, with consequences on the quality of life. Limited evidences showed a potential involvement of gut fungal communities. Here, the gut bacterial and fungal microbiota of a cohort of IBS patients have been characterized and compared with that of healthy subjects (HS). The IBS microbial community structure differed significantly compared to HS. In particular, we observed an enrichment of bacterial taxa involved in gut inflammation, such as *Enterobacteriaceae*, *Streptococcus*, *Fusobacteria*, *Gemella*, and *Rothia*, as well as depletion of health-promoting bacterial genera, such as *Roseburia* and *Faecalibacterium*. Gut microbial profiles in IBS patients differed also in accordance with constipation. Sequence analysis of the gut mycobiota showed enrichment of *Saccharomycetes* in IBS. Culturomics analysis of fungal isolates from feces showed enrichment of *Candida* spp. displaying from IBS a clonal expansion and a distinct genotypic profiles and different phenotypical features when compared to HS of *Candida albicans* isolates. Alongside the well-characterized gut bacterial dysbiosis in IBS, this study shed light on a yet poorly explored fungal component of the intestinal ecosystem, the gut mycobiota. Our results showed a differential fungal community in IBS compared to HS, suggesting potential for new insights on the involvement of the gut mycobiota in IBS.

**Key points:**

• *Comparison of gut microbiota and mycobiota between IBS and healthy subjects*

• *Investigation of cultivable fungi in IBS and healthy subjects*

• *Candida albicans isolates result more virulent in IBS subjects compared to healthy subjects*

**Supplementary Information:**

The online version contains supplementary material available at 10.1007/s00253-021-11264-4.

## Introduction

Irritable bowel syndrome (IBS) is a highly prevalent, stress-related functional gastrointestinal (GI) disorder that exhibits different GI and neurological symptoms, such as abdominal pain, diarrhea, constipation, and mood disorders (Ishihara et al. 2013). The gut microbiota plays an important role in modulating the communication between the central nervous system (CNS) and the GI tract, the so-called gut-brain axis (Sinagra et al. 2020). IBS is a clear example of a pathological condition involving alterations of this fine equilibrium between the gut microbiota and the CNS. The involvement of the gut microbiota in IBS physiopathology (Pinto-Sanchez et al. 2017) is suggested by the observation that probiotic treatments of IBS patients result in the relief of both GI and neurological symptoms. Furthermore, the gut barrier dysfunction widely demonstrated in IBS patients is strictly linked to microbial dysbiosis, also following into an altered modulation of the host immune system.

The IBS gut microbiota has been extensively studied. Antimicrobial peptides and some Toll-like receptors involved in bacterial recognition were shown to be upregulated in IBS (Ishihara et al. 2013; Simrén et al. 2013; Eisenstein 2016), and gut bacterial dysbiosis was investigated as a peripheral trigger. It has been shown that *Faecalibacterium prausnitzii* and *Bifidobacterium* genus were depleted in patients with IBS (Pittayanon et al. 2019).

On the other hand, fungi are still under investigation in IBS. Despite being a minor component of the gut microbial community, evidences indicated that fungi can play a role in gut diseases, as demonstrated in inflammatory bowel disease (Iliev et al. 2012; Richard et al. 2015; Sokol et al. 2017; Limon et al. 2017; Di Paola et al. 2020). Recent evidences, for example, suggested that fungi may partially act in the genesis of visceral hypersensitivity by activating the Dectin-1 receptor-mediated Syk/CARD9 signaling pathway (Chi et al. 2020).

Here, we characterized the gut microbiota (both bacterial and fungal communities) in IBS patients, taking into account also the two most common gastrointestinal symptoms related to gut motility, constipation, and diarrhea. Furthermore, we investigated the cultivable component of the gut mycobiota by characterizing the fungal isolates for their genotype and phenotype associated with GI tract resistance and potential virulence traits.

## Material and methods

### Subject recruitment

A total of 20 patients (13 males and 7 females, mean age 46.4 ± 6.3 years, range 37–60 years) with clinical diagnosis of IBS and 18 sex-matched healthy subjects (HS), as controls (10 males and 8 females, mean age 45.4 ± 5.8 years, range 18–50 years) were enrolled to the study (Supplementary Table S1).

The study protocol was approved by the Institutional Review Board of the Department of Gastroenterology, Piacenza Hospital (Italy). All participants, recruited at clinical study sites in Piacenza (Department of Gastroenterology, Hospital of Piacenza, Italy), signed informed consent. Inclusion criteria for IBS patient enrollment were in accordance with the Rome III criteria (Longstreth et al. 2006), providing standard conditions for diagnosis of IBS within a 12-week period, such as the presence of recurrent abdominal pain or discomfort at least 3 days/months in the last 3 months (12 weeks), associated with ≥ 2 of the following criteria: (i) improvement with defecation, (ii) onset associated with a change in stool frequency, (iii) onset associated with a change in stool form (appearance). The criteria are fulfilled with symptoms onset 6 months prior to diagnosis. All the subjects followed a Mediterranean-based diet and they did not take antibiotics or probiotics in the 3 months prior to sample collection.

### Fecal sample collection and total genomic DNA extraction

Fecal samples were collected at one time point, and subjects were carefully instructed on the method for sampling. Stool samples were stored at 4 °C until refrigerated transport (within 2 h) to the laboratory. An aliquot was used for fungal cultivation, while another one was used for bacterial and fungal genomic DNA extraction, then stored at – 80 °C.

Total DNA extraction from fecal samples (250 mg, wet weight) was performed using the FastDNA™ SPIN Kit for Feces (MP Biomedicals, Santa Ana, CA, USA), following the manufacturer’s instructions.

### Amplified ribosomal DNA restriction analysis

From each fecal sample (IBS and HS), bacterial 16S rDNA was amplified by PCR. DNA amplification was carried out in 25 μl PCR mixture containing 1× of PCR buffer, 2 mM of MgCl_2_, 200 μM of dNTPs, 0.4 μM of the primer 8F (5′-AGAGTTTGATCCTGGCTCAG-3′) and 1510R (5′-CGGTTACCTTGTTACGACTT-3′), 2.5U of *Taq* polymerase, and 10 ng of gDNA as template. PCR amplicons (10 μl) were digested with the *Hae*III, *Alu*I, *Rsa*I, and *Msp*I restriction enzymes (0.5 U/μl; Thermo Fisher Scientific, Milan, Italy) at 37 °C for 4 h. Restriction products were separated by electrophoresis, using 2.5% of agarose gel in 1× TAE buffer at 80 V for 3 h and visualized with 0.5-μg/ml ethidium bromide staining. A 1-kb ladder (GeneRuler 1 kb DNA ladder, Thermo Fisher Scientific, Milan Italy) was used as a DNA marker.

In order to compare the restriction patterns of each fecal sample, a binary matrix was constructed by using 0 and 1 values, as a function of the absence and presence of fragments in each restriction profile, respectively. Such matrix has been used for the construction of a dendrogram, based on amplified ribosomal DNA restriction analysis (ARDRA) patterns, by calculation of samples’ distance similarity according to the Jaccard Index (Levandowsly and Winter 1971) by mean of the “vegdist” function within the vegan R package (Oksanen et al. 2020). Hierarchical clustering according to the UPGMA method was performed by using the hclust function within the stats R package. Furthermore, unrooted neighbor-joining phylogenetic trees have been obtained by using the software package PHYLIP (Phylogeny Inference Package) (Plotree and Plotgram 1989).

### 16S rRNA gene and internal transcribed spacer 1 amplicon preparation, Illumina sequencing, and data analysis

Primer sets specific for the V3–V5 hypervariable regions (Fw: 5′TCGTCGGCAGCGTCAGATGTGTATAAGAGACAGCCTACGGGNGGCWGCAG; Rev: 5′GTCTCGTGGGCTCGGAGATGTGTATAAGAGACAGGACTACHVGGGTATCTAATCC3′) and for internal transcribed spacer 1 (ITS1), corresponding to ITS1F (nt 1761–nt 1779) 5′TCCGTAGGTGAACCTGCGG3′ and ITS4R (nt 2390– nt2409) 5′TCCTCCGCTTATTGATATGC3′, were used for amplification of the bacterial 16S rDNA gene and the fungal ITS1 rDNA region, respectively.

The gene amplicons were then purified and pair-end sequenced on Illumina MiSeq platform (IGA Technology Services, Udine, Italy) using a 2 × 300 nucleotide-paired reads protocol.

The raw fastq files were submitted to the European Nucleotide Archive with accession number PRJEB38069 (http://www.ebi.ac.uk/ena/data/view/ PRJEB38069) for 16S rDNA and with accession number PRJEB42596 for ITS1.

Reads were pre-processed using the MICCA pipeline (v. 1.5) (http://www.micca.org) (Albanese et al. 2015). Forward and reverse primer trimming and quality filtering were performed using *micca trim* and *micca filter*, respectively. De novo greedy clustering and chimera filtering were performed by using *micca otu*: operational taxonomic units (OTUs) were assigned by clustering the sequences with a threshold of 97% pairwise identity, and their representative sequences were taxonomically classified using *micca classify* with the RDP classifier v 2.11 on 16S rRNA gene sequencing data (Wang et al. 2007) and against the UNITE fungal ITS database (Kõljalg et al. 2013) on fungal ITS data. Multiple sequence alignment (MSA) of 16S rDNA sequences was performed using the Nearest Alignment Space Termination (NAST) (DeSantis et al. 2006) algorithm implemented in *micca msa* with the template alignment clustered at 97% similarity of the Greengenes database (DeSantis et al. 2006) (release 13_05). For fungal ITS1 sequences, de novo MSA was performed using MUSCLE (Edgar 2004). The phylogenetic trees were inferred using *micca tree* (Price et al. 2010). Sampling heterogeneity was reduced rarefying samples at the depth of the less abundant sample using *micca tablerare*. Before rarefaction, two samples with less than 2000 reads from the ITS1 dataset have been removed. Alpha (within-sample richness) and beta-diversity (between-sample dissimilarity) estimates were computed using the phyloseq R package (McMurdie and Holmes 2013). Permutational analysis of variance (PERMANOVA) test was performed using the adonis() function in the R package vegan with 999 permutations. Linear discriminant effect size analysis (LEfSe) was performed to find features (microbial taxa) most likely to explain differences between classes (Segata et al. 2011). All statistical analyses were performed using R (R CoreTeam 2012); *p*-values were false discovery rate (FDR)–corrected and considered significant with *p *< 0.05 (Benjamini and Hochberg 1995).

### Characterization of cultivable fungal isolates from fecal samples

Isolation and identification of fungal cultivable single-cell pure colonies from stool sample of the two cohorts of IBS patients and HS were performed as previously described by Strati et al. (2016).

### Phenotypical characterization of fungal isolates

Phenotypical traits that could be related to the ability of fungal isolates to survive/colonize the human gut have been studied. Growth at supra-optimal temperatures, pH impact on growth, resistance to bile acids, invasive growth, and hyphal formation have been tested for each fungal isolates, according to the methods described by Strati et al. (2016b).

Biofilm test was performed on YPD (Qvirist et al. 2016) on a flat-bottom 96-well plate by adding 200 μl of a fungal cell suspension equal to ~ 10^5^ colony forming unit (CFU)/mL in each well. The plate was incubated at 37 °C for 48 h. After the incubation period, cell suspensions were aspirated and each well with the adhered fungal cells was washed three times with deionized H_2_O and one time with PBS 1×. Biofilm-coated wells were then incubated with 0.01% of crystal violet (Sigma-Aldrich, Milan, Italy) for 30 min and washed as above. Finally, each well of the dried microtiter plate was incubated with 100 μl of 100% EtOH for 10 min and biofilms quantified by optical density measurement at 570 nm with a microplate reader (Synergy2BioTek, Winooski, VT, USA).

### Antifungal susceptibility testing

*Candida* isolates were tested for the susceptibility to the antifungal fluconazole and 5-flucytosine by measuring minimal inhibitory concentration (MIC), according to the Clinical and Laboratory Standards Institute (CLSI) (CLSI 2017). Antifungal resistance was analyzed according to CLSI clinical breakpoints (Pfaller and Diekema 2012; Castanheira et al. 2014).

### Random amplification of polymorphic DNA fingerprinting and phylogenetic analysis

*Candida albicans* isolates were genotyped by random amplification of polymorphic DNA (RAPD), using the primer Oligo-2 (5′-TCACGATGCA-3′) as previously described (Binelli et al. 2006). PCR conditions were 5 min at 94 °C, 30 s at 94 °C and 30 s at 36 °C for 40 cycles, and 2min at 72 °C, followed by a final extension of 10 min at 72 °C. DNA amplification was carried out in 25 μl PCR mixture containing 1× PCR buffer, 2 mM MgCl_2_, 200 μM of dNTPs, 0.4 μM of primer 2.5U of *Taq* polymerase, and 10 ng of gDNA as template. Amplification products were separated using a 1.5% of agarose gel in 1× TAE buffer at 80 V for 2 h and visualized with 0.5-μg/ml ethidium bromide staining. A 100–5000-bp ladder (GeneRuler™ Express DNA Ladder, Thermo Fisher Scientific, Milan, Italy) was used as a DNA marker.

A binary matrix was constructed by using 0 and 1 values, as a function of the absence and presence of fragments in each position of the gel for each RAPD profile. Such matrix has been used for the calculation of samples’ distance similarity according to the Jaccard Index (Levandowsly and Winter 1971) by mean of the “vegdist” function within the vegan R package and clustered hierarchically according to the UPGMA method by using the hclust function within the stats R package. Furthermore, unrooted neighbor-joining phylogenetic trees have been obtained by using the software package PHYLIP (Phylogeny Inference Package) (Plotree and Plotgram 1989).

### Statistical analysis

Wilcoxon rank-sum tests and Spearman’s correlations were performed using the R software (R CoreTeam 2012) through the stats R package (version 3.1.2) and the psych R package, respectively. All *p*-values have been corrected for multiple hypothesis testing controlling the false discovery rate (Benjamini and Hochberg 1995). Statistical significance of hematological parameters between the two cohorts was determined by Student’s *T* test (*p*-value < 0.05).

## Results

### The IBS gut microbiota showed dysbiotic profiles and bacterial markers associated with constipation

In order to explore the gut microbiota composition in IBS, we performed preliminary analyses based on hierarchical clustering of restriction profiles, obtained by amplified ribosomal DNA restriction analysis (ARDRA) on total DNA extracted from each fecal sample. ARDRA profiles showed a different cluster distribution of the IBS samples compared to HS (Supplementary Fig. S1A and S1B).

**Fig. 1:**
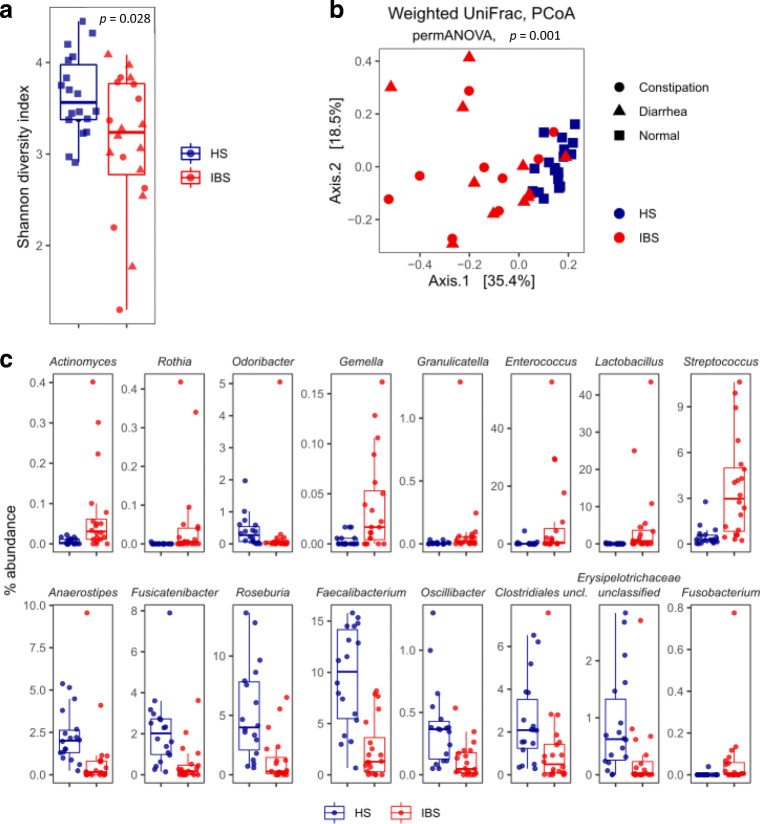
Characterization of the bacterial community structure in IBS patients and HS. **a** Alpha diversity, as measured by Shannon index; *p < 0.05, Wilcoxon rank-sum test. **b** Beta-diversity analysis performed with PCoA ordination based on the weighted UniFrac distances. **c** Relative abundances of the significantly different bacterial genera between IBS and HS (Wilcoxon rank-sum test; FDR-corrected *p* < 0.05)

Therefore, we performed a metataxonomic analysis of bacterial microbiota to better characterize the microbial community structure associated with IBS. Analysis of alpha diversity showed a significant reduction of bacterial richness in IBS compared to HS (Fig. 1a). The microbial community structure, as measured by beta-diversity analysis, was significantly different among IBS and HS (PERMANOVA, *p* < 0.01; Fig. 1b). All the hematological parameters (Supplementary Table S1), including ESR that was significantly higher in IBS group than HS (Student’s *T* test, *p* value < 0.01), as well as other variables (i.e., gender, symptoms etc.), did not affect the overall composition of the gut bacterial and fungal communities (PERMANOVA, *p* > 0.05).

The taxonomic analysis showed enrichment and depletion of several bacterial taxa (Wilcoxon rank-sum test FDR *p* < 0.05, Fig. 1c), as further confirmed by LEfSe (linear discriminant analysis effect size) analysis (*p* < 0.05, LDA > |2|; Supplementary Fig. S2). Unlike previous findings (Clemente et al. 2012), we did not observe differences in the *Firmicutes* to *Bacteroidetes* ratio between IBS and HS (Wilcoxon rank-sum test, *p* = 0.82). We found a significant enrichment of *Actinomyces*, *Rothia*, *Gemella*, *Streptococcus*, *Enterococcus*, *Granulicatella*, *Fusobacterium*, *Escherichia/Shigella*, *Veillonella*, and *Clostridium* cluster XI, and depletion of *Anaerostipes*, *Fusicatenibacter*, *Odoribacter*, *Oscillibacter*, *Roseburia*, and *Faecalibacterium* in IBS compared to HS (Fig. 1c, Supplementary Fig. S2).

Furthermore, we evaluated whether two common GI symptoms in IBS, diarrhea, and constipation, differentially contribute to the gut microbiota composition in IBS patients. We observed a significant enrichment of *Fusobacteria*, *Clostridium* cluster XVIII, and *Gemella* in constipated IBS patients (LEfSe, *p* < 0.05, LDA > |2|; Supplementary Fig. S3).

### Analysis of the gut mycobiota in IBS patients shows enrichment of *Saccharomycetes* and an overgrowth of *C. albicans* isolates

**Fig. 2:**
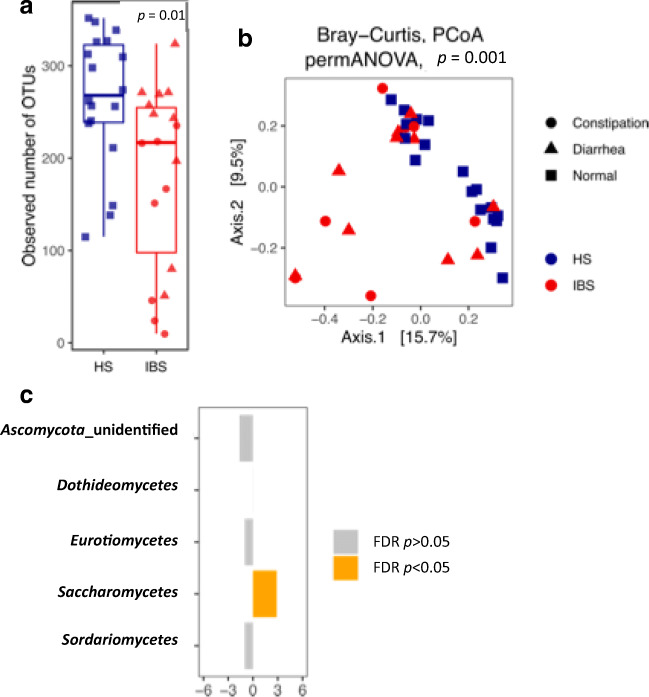
Characterization of the fungal community structure in IBS patients and HS. **a** Alpha diversity, as measured with observed number of OTUs; *p < 0.05, Wilcoxon rank-sum test. **b** Beta-diversity analysis performed with PCoA ordination based on the Bray-Curtis dissimilarity. **c** Welch’s *t* test statistics of the relative abundances of the gut mycobiota at family level in IBS (right) and HS (left) subjects. Orange bars indicate significant FDR-corrected *p* values adjusted for multiple comparison controlling the family-wise type I error rate

Exploration analyses of alpha and beta-diversity of the gut mycobiota showed a decrease of the observed fungal OTUs in IBS compared to HS (Fig. 2a, *p* value = 0.01) and a significant separation of IBS and HS samples in PCoA ordination based on Bray-Curtis distances (Fig. 2b, PERMANOVA, *p* value = 0.001). In IBS group, constipation and diarrhea did not affect the samples’ distribution. Metataxonomic analysis of the fungal communities showed an enrichment of *Saccharomycetes* in IBS (Fig. 2c, FDR-corrected *p* < 0.05, Welch *t* test).

**Fig. 3:**
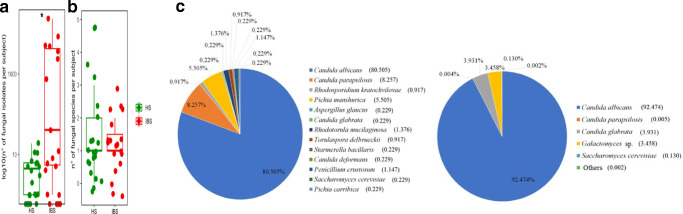
Cultivable gut mycobiota in IBS and HS groups. **a** Abundance (log10 number of isolates) and **b** richness of fungal species isolated from stool samples in HS and IBS groups (*p < 0.05, Wilcoxon rank-sum test). **c** Pie charts of the percentage of fungal species identified in (left) HS and (right) IBS

We further investigated the gut mycobiota composition of IBS and HS groups by characterizing the cultivable fungal isolates from fecal samples. We observed a significantly higher number of cultivable fungi (Fig. 3a, *p* = 0.028, Wilcoxon rank-sum test), as well as a trend of increase of fungal species richness (*p* = 0.07, Wilcoxon rank-sum test; Fig. 3b), in IBS compared to HS.

In both cohorts, *C. albicans* was the predominant isolated species, representing the 92.7% and the 80.5% of the fungal population in IBS and HS, respectively. *Candida glabrata* was the second most abundant species isolated in IBS, followed by *Galactomyces*. On the other hand, the gut mycobiota of HS group showed a greater diversity compared to IBS, with *Candida parapsilosis* being the second most represented fungal species in HS, in accordance with findings of Forbes et al. (2019). Furthermore, we found *Aspergillus*, *Rhodotorula*, *Penicillium*, and *Pichia* genera, known as commensals of diverse body sites (Rizzetto et al. 2014), and fungal species commonly used in food fermentation, such as *Torulospora delbrueckii* and *Starmerella bacillaris* (Bely et al. 2008; Englezos et al. 2015), exclusively in HS (Fig. 3c).

### *C. albicans* isolates from IBS are genetically and phenotypically distinct from HS isolates

**Fig. 4:**
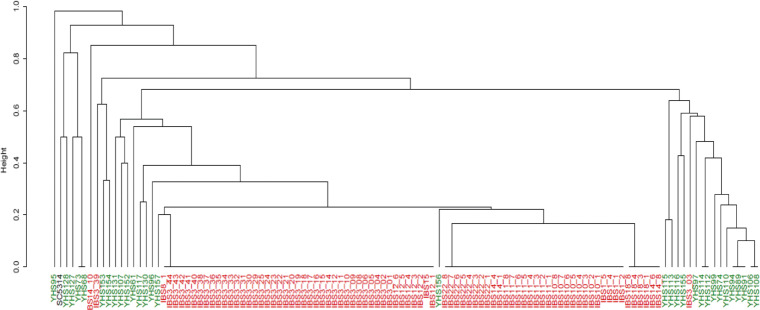
Cladogram representation of hierarchal clustering of *C. albicans* isolates from IBS and HS, based on RAPD profiles**.** Hierarchical clustering was obtained on calculated Jaccard distances by using UPGMA method. Unrooted neighbor-joining phylogenetic trees were obtained by the PHYLIP software package. *C. albicans* isolates from HS and IBS subjects were colored in green and red, respectively.

The aforementioned results indicated *C. albicans* as the most abundant species isolated from both IBS and HS. Therefore, we investigated the genetic diversity among these fungal isolates. RAPD genotyping (see “Materials and methods”) of *C. albicans* isolates clearly showed that IBS isolates are genetically unrelated to HS isolates. It is worth to note a clonal expansion of *C. albicans* isolates within some IBS patient (Fig. 4).

**Fig. 5:**
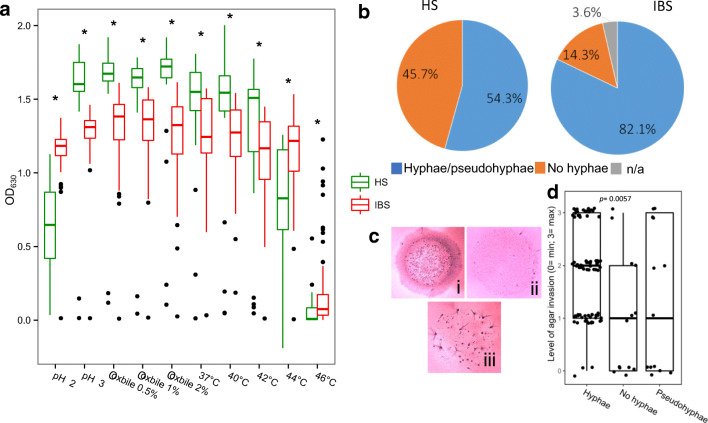
In vitro tests of resistance to GI-like and stressful conditions of *C. albicans* isolates. **a** Box plot representation of the growth of different *C. albicans* isolated in the present study (HS, green; IBS, red), at different GI-like and stressful conditions (such as pH, presence of bile acid, supra-optimal temperatures) as indicated by OD 630 nm (*FDR-corrected *p* < 0.0005 for Wilcoxon rank-sum test). **b** Ability to produce hyphae or pseudohyphae. Pie charts represent the percentage of isolates able to form hyphae or pseudohyphae in both the cohorts. **c** Invasiveness test. Growth of fungal colonies in agar medium and evaluation of the different degrees of invasiveness of fungal isolates: (i) highly invasive colony; (ii) not invasive colony; (iii) minimally invasive colony. **d** Correlation between hyphal formation and invasiveness of *C. albicans* isolates

Fungi can be passengers, commensals, or transient colonizers of the human GI tract (Rizzetto et al. 2014; Strati et al. 2016; Huseyin et al. 2017). Since stressful conditions (e.g., such as temperature, pH, and presence of bile acids) in the gut ecosystem can influence the microbial community status (Wang et al. 2014), we tested the ability of fungal isolates to resist under GI-like conditions or extreme stress in vitro. We found that *C. albicans* isolates from IBS showed lower growth in respect to HS isolates at the following growth conditions: at pH 3, in the presence of bile acids (0.5%, 1%, and 2%), and at supra-optimal temperatures (37 °C, 40 °C, and 42 °C). On the contrary, *C. albicans* isolates from IBS patients showed higher growth in respect to HS isolates at the following growth conditions: at pH 2 and at supra-optimal temperatures (44 °C and 46 °C) (*p* < 0.05; Fig. 5a). Furthermore, we tested virulence-related traits, such as the ability to produce hyphae (Fig. 5b) and invade the agar (Fig. 5c). We observed that most *C. albicans* isolates from IBS were able to produce hyphae (82.1%), while most *C. albicans* isolates form HS (45.7%) were not. Moreover, we observed that *C. albicans* isolates able to produce hyphae were more invasive than *C. albicans* isolates unable to produce hyphae in vitro (FDR-corrected *p* < 0.01, Wilcoxon rank-sum test) (Fig. 5d). IBS and HS subjects’ isolates, both able and unable to produce hyphae, were tested for the ability to form biofilm. We did not find significant differences among isolates of the two cohorts (Supplementary Fig. S4). Finally, we compared the susceptibility of *C. albicans* isolates to the most common antifungals, such as fluconazole and the non-azole antifungal 5-flucytosine (Table 1). As indicated by MIC90 values, *C. albicans* isolates from IBS were more susceptible to fluconazole than HS isolates.

**Table 1:** Antifungal resistance of *Candida* isolates from HS or IBS subjects

Species (no. of isolates HS-IBS)	Antifungals	Healthy subjects (HS)					IBS subjects				
		MIC (μg/ml) clinical breakpoints									
		MIC_50_	MIC_90_	%S	%SDD	%R	MIC_50_	MIC_90_	%S	%SDD	%R
*Candida albicans* (35–83)	Fluconazole	0.5	> 64	71.4	0	28.6	0.125	32	86.8	2.4	10.8
	5-Flucytosine	0.125	0.5	94.3	0	5.7	0.125	0.125	100	0	0
*Candida parapsilosis* (6–6)	Fluconazole	0.125	0.5	100	0	0	0.125	0.125	100	0	0
	5-Flucytosine	0.125	0.125	100	0	0	0.125	0.125	100	0	0
*Candida glabrata* (1–4)	Fluconazole	0.125	0.125	0	100	0	32	32	0	100	0
	5-Flucytosine	0.125	0.125	100	0	0	0.125	0.125	100	0	0

*S*, susceptible; *SDD*, susceptible dose-dependent or intermediate; *R*, resistant; *MIC*, minimal inhibitory concentration ranges: fluconazole, 0.125–64 μg/ml; 5-flucytosine, 0.125–64 μg/ml

## Discussion

The gut microbiota composition and its role in IBS pathophysiology have received great interest from the scientific community (Drossman 2016; Putignani et al. 2016). Its potential involvement in barrier dysfunction and visceral hypersensitivity is now widely recognized (Ishihara et al. 2013; Simrén et al. 2013; Piche 2014; Eisenstein 2016). The presence of bacterial dysbiosis associated to IBS has been confirmed by different metataxonomic studies (Collins 2014; Bhattarai et al. 2017; Li et al. 2019). A higher abundance of *Enterobacteriaceae* has been found in IBS patients when compared to healthy controls. Bacteria belonging to *Enterobacteriaceae* family, having the potential ability to invade intestinal epithelial cells or able to exacerbate gut inflammation, were observed in association with inflamed gut mucosa (Øyri et al. 2015).

The role of gut mycobiota in IBS was largely ignored so far. There are evidences about its involvement in colitis (Iliev et al. 2012), in particular in IBD (Sokol et al. 2017; Di Paola et al. 2020) and, not last, in stress-induced visceral hypersensitivity (Botschuijver et al. 2017). It has also been reported that some metabolic products of yeast metabolism could lead to symptoms related to IBS, and that fungal β-glucans of the cell wall that are normally recognized by the host immune system can induce visceral hypersensitivity (Botschuijver et al. 2017; Gayathri et al. 2020) and differential immune responses (Rizzetto et al. 2016; Di Paola et al. 2020).

In the present work, the explorative characterization of the intestinal microbial community structure by the ARDRA confirmed that the gut microbiota of IBS patients differs from HS, as previously described (Carroll et al. 2012; Vich Vila et al. 2018). Moreover, metataxonomic analysis showed a depletion of bacterial genera characterized by anti-inflammatory properties, such as *Roseburia* and *Faecalibacterium* and enrichment of well-known bacteria found involved in chronic gut inflammation, such as *Fusobacterium* (Bashir et al. 2016). The differential bacterial community structure in IBS compared with healthy controls, as observed in the present study, was in accordance with previous evidences (Tap et al. 2017).

In the gut ecosystem, fungi and bacteria coexist and directly interact with each other (Lynch and Pedersen 2016). Treatment with antibiotics in mice leads, on one hand, to the inhibition of bacteria and, on the other hand, to fungal expansion. This unbalance is then readily reverted following antibiotic cessation (Dollive et al. 2013). In an animal model of stress-induced IBS-like visceral hypersensitivity, fungicide treatment reduced visceral hypersensitivity in rat that has undergone maternal separation. Moreover, fecal transplantation in maternal separated rats showed mycobiome dysbiosis compared to non-handled rats, as controls (Botschuijver et al. 2017).

In humans, Das et al. (2021) showed that the mycobiome composition differentiates patients with IBS from controls, with a significant co-variation between mycobiota and bacteriome. Sokol et al. (2017) showed increased inter-microbial kingdom relationship in ulcerative colitis, and reduced interactions between gut bacteria and fungi in Crohn’s disease, while results from Imai et al. (2019) showed the contrary. Although the results were controversial, these studies indicated that unbalanced microbial network may play a crucial role in the gut inflammation.

All the above evidences were obtained by using next-generation sequencing technologies that allow to explore the complexity of the gut microbial ecosystem with high resolution. In the present study, we combined culture-independent (metagenomics) and culture-based approaches (culturomics) to the study of the gut fungal community. On the one hand, amplicon-based ITS1 sequencing allows for the description of fungal community composition, detecting the not-cultivable fungal population at high taxonomic level. On the other hand, culture-based analysis allows for discerning fungal phenotypes (e.g., virulence-related traits or antifungal resistance) that would be otherwise lost by metagenomics. Development of markers targeting pathogenicity traits, including markers detecting resistance to antifungals is fundamental to discriminate the “healthy” mycobiota from an altered one, often associated to disease conditions. Both methodologies are valuable, especially if combined together. In the present work, sequencing analysis showed a limited ability to identify specific taxa more than order level. We identified enrichment of *Saccharomycetes* in IBS compared HS. In general, fungal metagenomics procedure suffers from some limitation, especially due to the wide variance in the mycobiome taxonomic composition, as highlighted by Das et al. (2021), which suggest to consider sequencing both the ITS1 and ITS2 regions.

By using the culturomics approach, we confirmed an imbalanced fungal community in accordance with results from Gu et al. (2019) and Das et al. (2021). In both our cohorts, the most abundant fungal species was *C. albicans*, isolated at higher frequency in IBS patients than HS. This fungal species is generally recognized as human colonizer, inhabiting several body districts (Rizzetto et al. 2014). Also, Hong et al. (2020) found *Candida* as the most abundant fungal genus in IBS patients, positively correlated to severity of bloating and anxiety.

Previous evidences reported that intestinal fungal dysbiosis promote visceral hypersensitivity via the Dectin-1/Syk signaling pathway (involved in controlling of the systemic fungal infection) in IBS patients and rats (Botschuijver et al. 2017; Taylor et al. 2007). Moreover, candidalysin, a cytolytic peptide toxin produced by *C. albicans*, has been demonstrated to damage epithelial barrier and to induce mucosal inflammation (Kasper et al. 2018).

These results highlight a critical role of *Candida* in the pathophysiology of IBS. In our study, the phenotypical characterization of *C. albicans* isolates revealed that the isolates from IBS were significantly more resistant to in vitro stress conditions than HS ones, but they were less resistant when challenged by GI-like conditions. It is known that *C. albicans* changes the expression of specific pH-related genes to inhabit tissues with different environmental pH (Mühlschlegel and Fonzi 1997; Calderone and Fonzi 2001) and carries out strategies to become more virulent by producing hyphae (Staib et al. 2000; Karkowska-Kuleta et al. 2009). In IBS patients, we found a high percentage of *C. albicans* isolates able to form hyphae, and to invade the agar medium, in accordance with previous observations (Felk et al. 2002). Thus, we could hypothesize that fungal isolates with peculiar virulent and invasive traits could contribute to visceral hypersensitivity associated to IBS.

Moreover, we discover differences in the genotypic background of *C. albicans* isolates between IBS and HS. Hierarchical clustering showed that IBS isolates present high genotypic relatedness among them with respect to HS isolates. We could speculate a putative clonal expansion of *C. albicans* strains in IBS, similar to our previous observations for *Saccharomyces cerevisiae* isolates from IBD patients (Ramazzotti et al. 2019). In this previous study, we hypothesized that in the human intestine, a clonal expansion of strains or multiple colonization of strains from the same source could occur. Both phenomena could indicate that a gut dysbiosis, occurring during inflammatory processes, could lead to the expansion or the survival of strains with peculiar characteristics contributing to gut symptoms.

In general, the specific mechanisms of the mycobiome involved in IBS yet remain to be clarified. Critical issue has been raised about the potential contribution of gut mycobiota analysis for diagnostic usefulness, although a pathogenetic importance of the mycobiota in IBS was recognized (Das et al. 2021).

Finally, we tested the different antifungal susceptibility of *Candida* spp. isolates (such as *C. albicans*, *C. parapsilosis*, and *C. glabrata*) from our cohorts, in order to evaluate presence of antifungal-resistant isolates in IBS. Over the last decade, the cases of infections by opportunistic pathogens, in particular of *C. albicans*, *C. parapsilosis* (Trofa et al. 2008), and *C. glabrata* (Fidel Jr et al. 1999), have increased greatly. It is now recognized that inappropriate antifungal use contributes to the increase in antifungal resistance, dramatically reducing the therapeutic panel and the strategies of eradication of pathogens (Sanglard and Odds 2002; Chen et al. 2010; Arendrup et al. 2011; Strati et al. 2016a). In general, our assays showed few differences in the susceptibility towards fluconazole and 5-flucytosine between *Candida* spp. isolates from IBS and HS. *C. glabrata* isolates from IBS, different from HS ones, were resistant to fluconazole, as previously described (Fidel Jr et al. 1999). *C. albicans* and *C. parapsilosis* isolates from IBS were less resistant to antifungals when compared to isolates from HS. More in general, we did not find substantial differences between IBS patients and healthy controls. However, because of increased resistance of pathogenic fungi, the classical fungicidal should be used with more caution for regular IBS therapy, although they are reserved for highly invasive and often lethal infections. Different strategies of mycobiota modulation may be appropriate for IBS, including the use of probiotics (fungal and bacterial strains) that are able to induce a favorable gut microbial immunomodulation.

Overall, our results on fungal isolate differences suggest that investigation at the strain level could be relevant for a better understanding of the role of the gut microbial community in IBS. The gut mycobiota may be involved in IBS and contribute to intestinal hypersensitivity as much as the bacterial counterpart. Future research should be addressed to investigate the potential immunomodulation of IBS fungal isolates, as previously demonstrated in IBD (Di Paola et al. 2020), and to evaluate whether bacterial and fungal dysbiosis could act in concert during the gut inflammation in IBS.

## Supplementary information

ESM 1 (PDF 451 kb)
