# Supplementary material for: Gut microbiota profiles and characterization of cultivable fungal isolates in IBS patients
Source: Appl Microbiol Biotechnol. 2021 Apr 10;105(8):3277–88. doi: 10.1007/s00253-021-11264-4 (PMC8053167; doi:10.1007/s00253-021-11264-4)
Supplement: Supplementary file 1 — (PDF 451 kb) [file 253_2021_11264_MOESM1_ESM.pdf]

**Applied Microbiology and Biotechnology**

# Gut microbiota profiles and characterization of cultivable fungal isolates in IBS patients

Piero Sciavilla[1*], Francesco Strati[2*], Monica Di Paola[3], Monica Modesto[1], Francesco Vitali[5], Duccio Cavalieri[3], Gian Maria Prati[4], Maura Di Vito[1], Giovanni Aragona[4], Carlotta De Filippo[5*], Paola Mattarelli[1**]

[1]Department of Agriculture and Food Sciences, University of Bologna, Viale Fanin 44, 40127 Bologna, Italy
[2]Laboratory of Mucosal Immunology, Department of Experimental Oncology, European Institute of Oncology, Via Adamello 16, 20139 Milan, Italy.
[3]Department of Biology, University of Florence, Via Madonna del Piano 6, 50019 Sesto Fiorentino, Florence, Italy
[4]Department of Gastroenterology and Hepatology, "G da Saliceto" Hospital, Via Taverna 49, 29121 Piacenza, Italy
[5]Institute of Agriculture Biology and Biotechnology, National Research Council (CNR), Via Moruzzi 1, 56124 Pisa, Italy

* These authors contribute equally to the work

[**]Corresponding author:
Paola Mattarelli, paola.mattarelli@unibo.itphone +39 0512096267

**Supplementary Table S1**. Characteristics of study participants and hematological parameters at the moment of fecal sampling.

| | **IBS subjects (n = 20)** | **HS subjects (n = 18)** |
|---|---|---|
| Age, years (mean ± SD) | 46.4 ± 6.3 | 45.4 ± 5.8 |
| Gender, male (n) | 13 | 10 |
| BMI (mean ± SD) | 22 ± SD | 23 ± SD |
| Smokers, (n) | 7 | 6 |
| ***Symptoms*** | | |
| Constipated | 11 (55%) | |
| Abdominal pain | 7 (35%) | |
| Bloating | 14 (70%) | |
| Flatulence | 4 (20%) | |
| Diarrhea | 9 (45%) | |
| ***Hematological parameters*** | | |
| White blood cell count (x $10^9$/L) | 5393.4 ± 1411.8 | 5700 ± 1310* |
| Creatinine (µmol/L) | 0.905 ± 0.2 | 0.7 ± 0.2 * |
| Urea (mg/dL) | 45.6 ± 5.8 | 46.4 ± 3.5 * |
| Cholesterol (mg/dL) | 188.2 ± 25.7 | 175.3 ± 23.6 * |
| Glucose (mg/dL) | 99.6 ± 14.7 | 91.5 ± 13 * |
| ESR (mm/h) | 10.3 ± 3.1 | 5.2 ± 2.2** |

BMI: body mass index; ESR: erythrocyte sedimentation rate. *, not significant ($p$>0.5); **, significant ($p$>0.01)

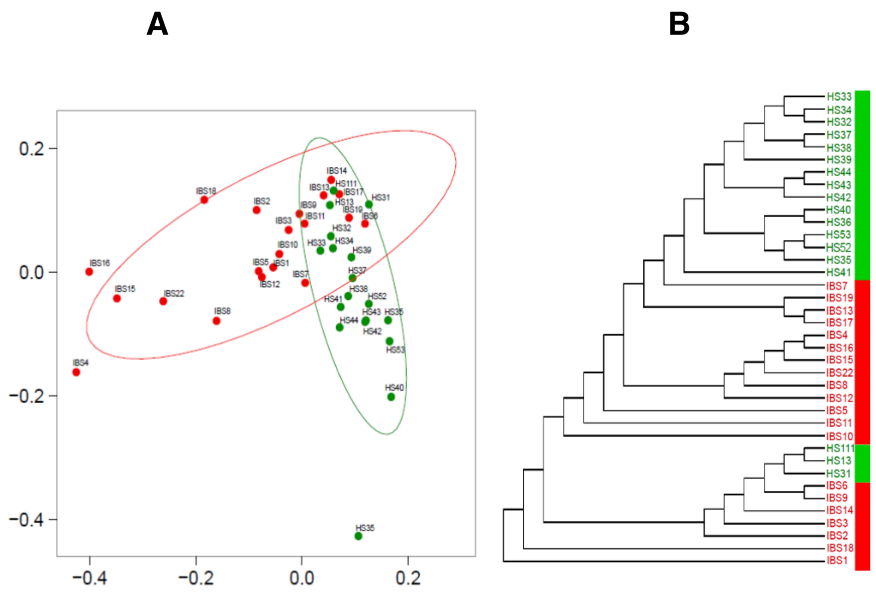


**Supplementary Fig. S1** Gut microbial community structure of the two studied cohorts by restriction profiles of microbial communities obtained by ARDRA. A) Sample distribution of IBS (red) and HS subjects (green), according to gut microbiota composition by Multidimensional scaling analysis and B) UPGMA hierarchical clustering calculated on Jaccard distances of the restriction profiles.

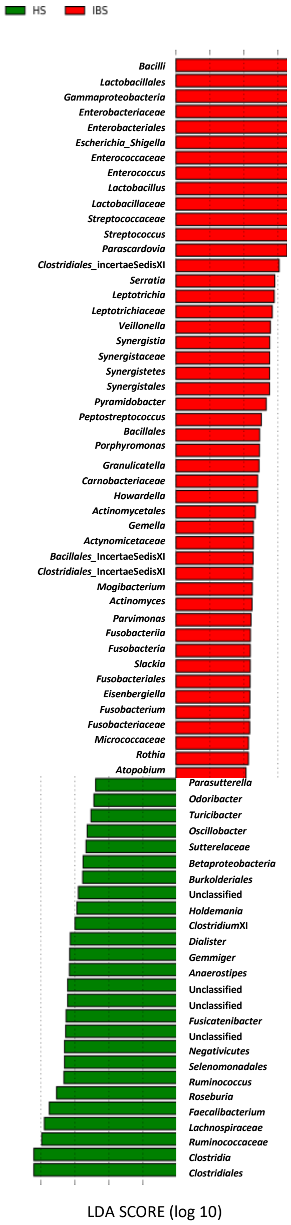


**Supplementary Figure S2.** Differences in bacterial taxa among IBS and HS. LEfSe analysis shows a statistically significant enrichment of bacterial genera in the two cohorts. LEfSe results indicate a sequentially significant ranking among groups (Alpha value = 0.05 for the factorial Kruskal–Wallis test among classes). The threshold for the logarithmic LDA score was 2.0.

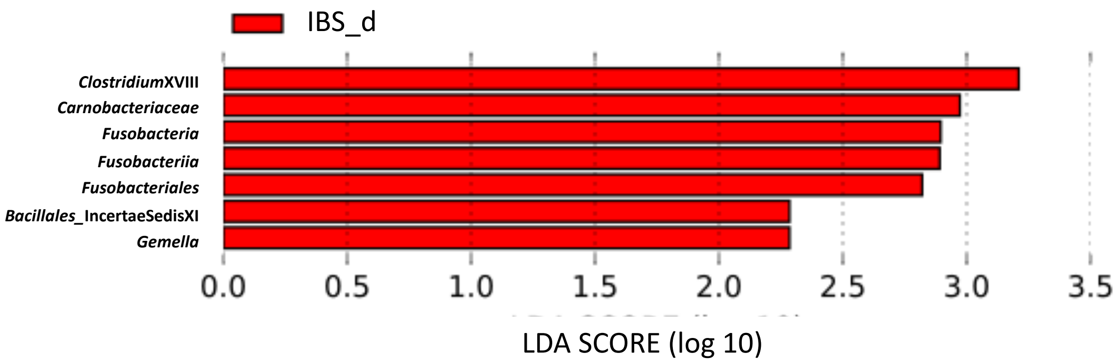


**Supplementary Fig. S3**. Differences in bacterial taxa among IBS patients with symptoms of constipation (IBS-C) or diarrhea (IBS-D). LEfSe analysis shows a statistically significant enrichment of bacterial genera. LEfSe results indicate a sequentially significant ranking among groups (Alpha value=0.05 for the factorial Kruskal–Wallis test among classes). The threshold for the logarithmic LDA score was 2.0.

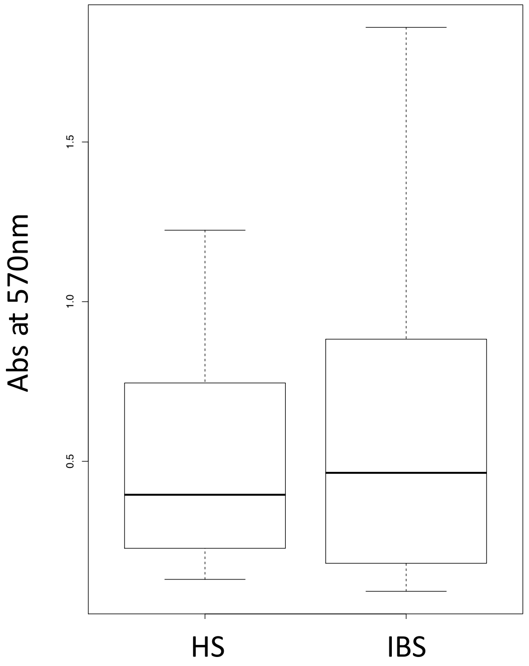


**Supplementary Fig. S4.** Box plot of biofilm formation by HS and IBS *C. albicans* isolates. Box plots are defined by the 25th and 75th percentiles. Center line represents the median (50th percentile). Whiskers are defined as 1.5 times the interquartile range (IQR, 75th–25th percentile), plus or minus the 75th and 25th percentiles, respectively. Y-axis, Abs (absorbance) at 570 nm.
